# Compared with intensity‐modulated radiotherapy, image‐guided radiotherapy reduces severity of acute radiation‐induced skin toxicity during radiotherapy in patients with breast cancer

**DOI:** 10.1002/cam4.1630

**Published:** 2018-07-03

**Authors:** Jang‐Chun Lin, Jo‐Ting Tsai, Yu‐Ching Chou, Ming‐Hsien Li, Wei‐Hsiu Liu

**Affiliations:** ^1^ Department of Radiation Oncology Shuang Ho Hospital Taipei Medical University Taipei Taiwan; ^2^ Department of Radiology School of Medicine College of Medicine Taipei Medical University Taipei Taiwan; ^3^ Graduate Institute of Clinical Medicine College of Medicine Taipei Medical University Taipei Taiwan; ^4^ School of Public Health National Defense Medical Center Taipei Taiwan; ^5^ Department of Neurological Surgery Tri‐Service General Hospital and National Defense Medical Center Taipei Taiwan; ^6^ Graduate Institute of Medical Sciences National Defense Medical Center Taipei Taiwan

**Keywords:** acute radiation‐induced skin toxicity, breast cancer, image‐guided radiotherapy

## Abstract

Radiotherapy (RT) is an effective treatment for breast cancer. The side effects of breast irradiation, including skin toxicity in the irradiation field, cause considerable discomfort. This study compared the severity of skin toxicity caused by image‐guided RT (IGRT) and intensity‐modulated RT (IMRT) combined with an electronic portal imaging device (EPID) in breast cancer. This study retrospectively analyzed 458 patients with breast cancer who had received RT. The patients were divided into two groups: 302 and 156 patients in the IMRT and IGRT groups. In the IGRT group, 8 and 148 patients had received helical tomotherapy irradiation and IMRT with cone‐beam computed tomography. Simple and multiple logistic regression analyses were used to estimate the relationship between RT technique and the severity of radiation skin toxicity. In our study, 284, 97, and 6 patients exhibited grades I, II, and III radiation dermatitis (RD). Moreover, 75 patients in the IMRT group (24.80%) and 22 patients in the IGRT group (14.10%) exhibited grade II RD. All patients with grade III RD were in the IMRT group (2.00%). No patient exhibited grade IV RD. The patients in the IGRT group exhibited less severity of RD than in the IMRT group. The severity of acute RD due to IGRT is significantly lower than that due to IMRT with EPID.

## 1 INTRODUCTION

Breast cancer is commonly detected in its early stage because of the extensive availability of mammography.^1^ The standard treatment for early stage breast cancer is conservative surgery combined with radiotherapy (RT) administered to the whole breast. Adjuvant whole‐breast RT provides increased local tumor control and considerably reduces the risk of death.^2^ Early stage breast cancer after breast‐conserving surgery (BCS) or advanced breast cancer after modified radical mastectomy (MRM) with positive lymph nodes^3^ should be treated using adjuvant RT, which can increase the local tumor control rate and overall survival.^4^ RT is a type of highly effective and targeted treatment used for destroying microscopic cancer cells that may have escaped surgical removal.^5^

The most undesirable side effect of breast irradiation is skin toxicity in the irradiation field. Potential inhomogeneity of radiation doses is an inherent drawback associated with a standard tangential beam arrangement for breast RT. The currently used conventional tangential technique has negative effects on long‐term cosmesis and has acute side effects.^6^ Intensity‐modulated RT (IMRT) is a more complex form of conformal RT than the conventional tangential technique.^7^ IMRT is more advanced than conventional techniques, and the equipment consists of a variable aperture to shape dose distributions around targets; these distributions cannot be achieved using conventional equipment. Research showed that potentially more suitable dose distributions in the clinical target volume (CTV) of the breast, a lower incidence of acute skin toxicity, and lower doses to normal lung or heart tissues were associated with IMRT than with the standard technique; in addition, the cosmetic results after IMRT use were excellent.^8^ Moreover, a prospective randomized trial comparing standard RT and IMRT reported that the breast appearance changed more visibly with standard RT than with IMRT in 233 patients with breast cancer.^9^

Image‐guided RT (IGRT) is a modality that uses imaging through computed tomography (CT) during RT to improve the accuracy and precision of radiation delivery. IGRT means that using TomoDirect, volumetric‐modulated arc therapy (VMAT), or IMRT planning techniques combined with CT image matching before RT delivery. It seems that IGRT has no difference in RT planning of skin dose or cardiac dose from IMRT. IGRT provides the possibility of reducing setup margins; this technique also effectively spares normal tissue while promoting dose escalation to the tumor. Therefore, the visualization of the surgical tumor bed as outlined by the lumpectomy cavity or the fiducial markers and the organs at risk (OARs) during RT is necessary. It may allow administration of a high radiation dose to the areas at risk of recurrence while reducing irradiation of normal organs such as the lungs and heart. Compared with the advantages associated with IMRT, TomoDirect could be a suitable radiation method for whole‐breast irradiation without nodal irradiation.^10^ Helical tomotherapy (TOMO) is also a tool of IGRT, which uses a megavoltage CT (MVCT) scan immediately prior to radiation treatment and verifies the setup error before RT. IGRT may attenuate radiation doses to small organs, such as the cochlea, considerably more effectively than IMRT does without sacrificing target coverage in patients with head and neck cancer.^11^ Another potential advantage of IGRT over IMRT is that the patients’ pattern of breathing‐related movements can be monitored during RT with pretreatment imaging, as observed with four‐dimensional cone‐beam CT (CBCT).

We recently reported that when modern radiation techniques are used, neither the prone nor supine position provides higher attenuation of radiation doses at the OARs than the other position. TOMO was determined to be superior to IMRT and VMAT in terms of sparing the OARs and planning quality parameters in rectal cancer.^12^ We conducted this study to observe differences in the severity of the radiation‐induced skin toxicity after IGRT (including TOMO and irradiation with CBCT) and IMRT used in combination with an electronic portal imaging device (EPID).

## 2 MATERIALS AND METHODS

Some patients received treatment for primary tumors after operation, whereas some did not. The treatment methods were approved by the Multidisciplinary Breast Tumor Board at Shuang Ho Hospital, Taipei Medical University. The patients received an explanation regarding the benefits, treatment durations, and possible complications of IGRT as well as of IMRT, and they were then asked to select a treatment modality. The patients included in the study were women aged 20‐85 years; had Eastern Cooperative Oncology Group (ECOG) performance status 0, 1, or 2; and had pathological proof of breast cancer either with or without surgery (BCS or MRM). The exclusion criteria for this retrospective study were as follows: (1) patients had a previous history of thorax RT, (2) unclear consciousness, (3) or ECOG performance status 3 or 4. We evaluated the patients for radiation‐related toxicity according to the Common Terminology Criteria for Adverse Events V3.0 (CTCAE V3.0).^13^

### 2.1 Patient characteristics

Table 1 summarizes the patient characteristics. From the Breast Cancer Registry of the Department of Radiation Oncology, Shuang Ho Hospital, we enrolled 458 patients with breast cancer who had received treatment between January 2012 and December 2014 and retrospectively reviewed whether the patients received IMRT or IGRT. We included the patients which received breast surgery with BCS or MRM, and even no previous surgery with biopsy only. The radiation dose would be used according to the rule of no breast surgery with 60‐70 Gy, BCS with 60 Gy, and MRM with 50 Gy. The median observation time for all patients was 45 days. The factors for analysis included age; smoking habit; surgery; tumor stage; irradiated target site, including the CTV; RT technique; total RT treatment time; radiation skin toxicity; radiation skin toxicity days (RSTD, defined as the time in which the patient exhibited radiation skin toxicity after beginning irradiation); and predictive breast molecular biomarkers including estrogen receptors, progesterone receptors, and human epidermal growth factor receptor‐2. Tumor staging was conducted according to the seventh edition of the American joint Committee on Cancer Criteria.

**Table 1:** The distribution of demography and clinical characteristics by RT techniques

	IMRT (n = 302)	IGRT (n = 156)	*P* value^a^
	n (%)	n (%)	
Age, M ± SD	54.59 ± 10.90	54.48 ± 10.70	.919
Smoke			
No	292 (96.70)	152 (97.40)	.780^b^
Yes	10 (3.30)	4 (2.60)	
ER			
No	60 (20.60)	38 (24.70)	.389
Yes	231 (79.40)	116 (75.30)	
N/A	11	2	
PR			
No	80 (27.50)	44 (28.60)	.896
Yes	211 (72.50)	110 (71.40)	
N/A	11	2	
Her‐2			
No	199 (70.80)	104 (68.00)	.612
Yes	82 (29.20)	49 (32.00)	
N/A	21	3	
Surgery			
Biopsy only	11 (3.60)	5 (3.20)	.461
BCT	211 (69.90)	118 (75.60)	
MRM	80 (26.50)	33 (21.20)	
Tumor stage			
Tis	44 (14.60)	28 (17.90)	.471
1	111 (36.80)	61 (39.10)	
2	111 (36.80)	55 (35.30)	
3	21 (7.00)	5 (3.20)	
4	15 (5.00)	7 (4.50)	
Nodal stage			
0	172 (57.00)	96 (61.50)	.393
1	76 (25.20)	30 (19.20)	
2	32 (10.60)	21 (13.50)	
3	22 (7.30)	9 (5.80)	
Metastatic stage			
0	294 (97.40)	149 (95.50)	.441
1	8 (2.60)	7 (4.50)	
AJCC stage			
0	46 (15.20)	28 (17.90)	.567
1	80 (26.50)	46 (29.50)	
2	107 (35.40)	48 (30.80)	
3	61 (20.20)	27 (17.30)	
4	8 (2.60)	7 (4.50)	
Irradiated target side			
Right	144 (47.80)	77 (49.40)	.083
Left	151 (50.20)	70 (44.90)	
Bilateral	6 (2.00)	9 (5.80)	
CTV			
Breast only	176 (58.30)	92 (59.00)	.547^b^
Chest wall only	9 (3.00)	4 (2.60)	
Breast + SCF	47 (15.60)	31 (19.90)	
Chest wall + SCF	70 (23.20)	29 (18.60)	

BCS, breast conserving surgery; CTV, clinical targeted volume; ER, estrogen receptors; HER‐2, human epidermal growth factor receptor‐2; IGRT, image‐guided radiotherapy; AJCC, American Joint Committee on Cancer; SCF, supraclavicular fossa; IMRT, intensity‐modulated radiotherapy; MRM, modified radical mastectomy; N/A, not applicable; PR, progesterone receptors; RT, radiotherapy.

a Independent *t* test or chi‐square test.

b Fisher's exact test.

### 2.2 Simulation, target definition, and dose prescription

For breast irradiation planning, images were acquired through spiral CT without intravenous contrast material. CT simulation images were acquired with each patient in the supine position. Customized devices were used, and the patient was immobilized using pillow vacuum bags (Klarity Medical Products, USA 1987 Coffman Road Newark, Ohio 43055 Peter M. Larson, President). Bilateral arms were abducted and immobilized at the same time above the patient's head using pillow vacuum bags. A skin line marker was used to set reference points, and all surgical scars were also marked with copper wire to identify tumor bed or original breast region. The CT‐simulated images got slice thickness of 5 mm from cervical spine level 3 to lumbar spine level 2.

According to the contouring guideline of The Radiation Therapy Oncology Group, the gross tumor volume included the gross breast tumor, if the patient had not received further surgery. The CTV was defined as the entire residual breast tissues after BCS or of the chest wall after MRM. The supraclavicular fossa field was irradiated irrespective of whether sentinel or axillary lymph nodes were invaded by cancer cells. The planning target volume (PTV) margins were determined by the physician, and they varied from case to case. Finally, PTV* was defined as the PTV excluding a skin depth of 3 mm and was determined by a medical physicist to prevent skin toxicity. The prescribed dose was 2 Gy in 25 fractions for a total dose of 50 Gy to the entire residual breast tissue or chest wall. Then, a boost dose of 10 Gy was delivered to the tumor bed postoperatively using the stick marker technique or 10‐20 Gy to the gross tumor. Radiation planning quality was asked to match a minimum dose of greater than 95% and a maximum dose of less than 110% of the prescribed dose. The OARs included the ipsilateral lung, whole lung, heart, trachea, and cervical esophagus. The constraints of the OARs obeyed the following rules according to Quantitative Analyses of Normal Tissue Effects in the Clinic (QUANTEC)^14^: the ipsilateral lung mean dose <7 Gy, the whole lung V30 < 20%, V20 < 30%, V10 < 40%, the whole lung mead dose <13 Gy, the heart V25 < 10%, the tracheal mean dose <44 Gy, and the esophagus mean dose <34 Gy.

### 2.3 TOMO technique

Tomotherapy plans were developed on the Tomotherapy Planning Station Hi‐Art^®^ Version 4.2.3 workstation (Tomotherapy Incorporated, Madison, WI, USA) with a superposition/convolution algorithm. Because of workstation limitations, CT contouring and OAR images were drawn in Version 9.2 of the Pinnacle3 planning system and transferred to the TOMO planning system. All CT contours were planned in the helical mode using a 6‐MV photon beam.^12^

### 2.4 IMRT technique

A 6‐MV photon beam with 6‐7 coplanar beams and CT‐based treatment planning (Pinnacle Version 9.2) was used. The doses were delivered using a linear accelerator equipped with multileaf collimators.^15^

### 2.5 Verification

**Figure 1:**
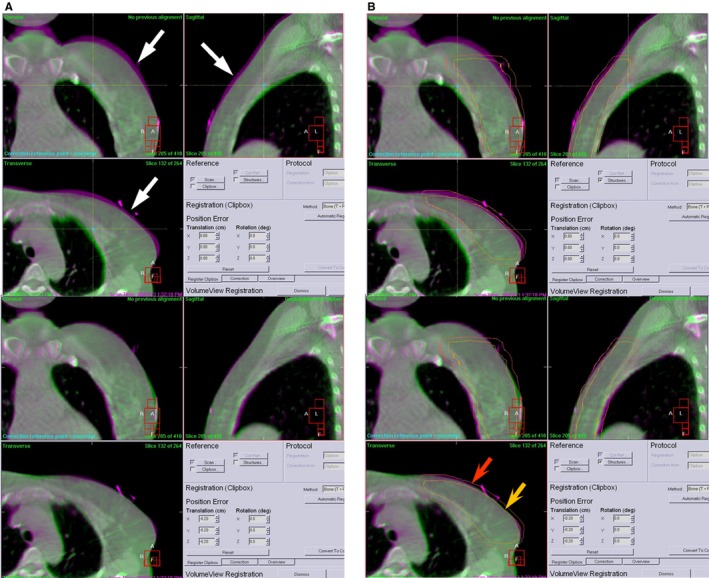
A, This breast CBCT image in upper before autofusion mapping; white arrow tip pointed out the predominant skin gap in pink color; after autofusion in lower, this skin gap could disappear. B, This breast CBCT image before autofusion mapping in upper; red arrow tip showed PTV; yellow arrow showed CTV irradiation field; after autofusion in lower, CTV can nearly matched breast CT image. CBCT, cone‐beam computed tomography; CTV, clinical target volume; PTV, planning target volume

We performed IGRT including CBCT and TOMO daily. The contours of the CTV, PTV, and lung were transmitted to the digitally reconstructed images created using virtual simulation. The setup processes were verified before radiation treatment for each patient by coinciding the skin center and room laser. An EPID was used to identify the accuracy of IMRT alone. In the IGRT group, we acquired CBCT or MVCT images at a minimum distance of 5 cm above and below the level of the PTV in the treatment position.^16^ In our study, extensive setup error was defined as 5 mm above the margin along any direction. The breast CBCT images were used for autofusion mapping depending on the CTV of the soft tissue. An example is illustrated in Figure 1A,B.

### 2.6 Endpoints and statistical analysis

Data were collected retrospectively from medical records. Skin toxicity according to the CTCAE V.3 grading system was the primary endpoint. The severity of radiation dermatitis (RD) was assigned according to the most severe grade of dermatitis on the whole breast/chest wall described in the medical records by a radiation oncologist. Smoking habit, clinical characteristics, and side effects are described as counts, percentages, and means ± standard deviations, and we evaluated the differences between RT techniques for each factor using the Pearson chi‐squared test and independent *t* test. Simple and multiple logistic regression analyses were used to estimate odds ratios (ORs) and 95% confidence intervals (CIs) for analyzing the associations between RT technique used and severity of radiation skin toxicity risk. IBM SPSS Statistics version 22.0 was used for all statistical analyses. All statistical tests were two tailed, and *P *<* *.05 indicated statistical significance.

## 3 RESULTS

We divided the patients into two groups. The IMRT group consisted of 302 patients; the IGRT group consisted of 156 patients including 8 patients who had undergone TOMO and 148 patients who had undergone RT and CBCT. The two groups did not differ significantly in any characteristic except for the severity of radiation‐induced skin toxicity. Three hundred and eighty‐seven patients exhibited radiation skin toxicity. According to the CTCAE V.3 grading system for evaluating and categorizing the severity of radiation‐induced skin toxicity, 284, 97, and 6 patients exhibited RD of grades I, II, and III, respectively. In total, 71 patients had no further skin toxicity after breast irradiation, of whom 48 and 23 belonged to the IMRT and IGRT groups, respectively. Grade II RD was observed in 75 patients in the IMRT group (24.80%) and 22 patients in IGRT group (14.10%). All 6 patients experiencing grade III RD had received IMRT. No patient exhibited radiation‐induced grade IV skin toxicity.

Among the patients who received RT, IGRT appeared to be superior to IMRT because it induced less severe skin toxicity (*P *=* *.006) than IMRT did; however, IGRT did not show any benefits over IMRT in terms of medicine usage after toxicity occurrence (*P *=* *.405) or RSTD (*P *=* *.747). The relationship between the side effects and RT techniques is presented in Table 2. Furthermore, the side effects were classified into two groups, namely grade 0 + grade I and grade II + grade III. The correlations among severity of radiation skin toxicity and other factors are shown in Table 3. According to our observation, irrespective of whether univariate (OR, 0.45; 95% CI, 0.27‐0.75; *P *=* *.002) or multivariate (OR, 0.48; 95% CI, 0.28‐0.82; *P *=* *.007) logistic regression was used, a significant difference was observed between the RT techniques and severity of RD. In other words, IGRT induced significantly lower skin toxicity after breast irradiation. The RT technique used was the only factor affecting the severity of the side effect, namely radiation‐induced skin toxicity. The effects of smoking habit, molecular biomarkers, disease stage, and type of surgery or irradiation field on skin toxicity did not differ between the two groups.

**Table 2:** The relationship between side effects and RT techniques

	IMRT (n = 302)	IGRT (n = 156)	*P* value^a^
	n (%)	n (%)	
RT side effect			
No	48 (15.90)	23 (14.70)	.852
Yes	254 (84.10)	133 (85.30)	
Gr.			
0	48 (15.90)	23 (14.70)	.006^b^
I	173 (57.30)	111 (71.20)	
II	75 (24.80)	22 (14.10)	
III	6 (2.00)	0 (0)	
RSTD (M ± SD; d)	35.70 ± 9.00	35.97 ± 7.71	.747
Medication			
No	82 (27.20)	36 (23.10)	.405
Yes	220 (72.80)	120 (76.90)	
TRTT (M ± SD; d)	45.14 ± 6.72	45.72 ± 5.40	.347

Gr., grade; IGRT, image‐guided radiotherapy; IMRT, intensity‐modulated radiotherapy; M ± SD, mean ± deviation; RSTD, radiation skin toxicity days; RT, radiotherapy; TRTT, total RT treatment time.

a Independent *t* test or chi‐square test.

b Fisher's exact test.

**Table 3:** The correlative factors’ analyses of radiation skin toxicity grade (Gr. II + III vs 0 + I)

Variables	Univariate		Multivariate	
	OR (95% CI)	*P* value	OR (95% CI)	*P* value
Age (y)	1.01 (0.99‐1.04)	.238	1.01 (0.99‐1.04)	.238
RT techniques (IGRT vs IMRT)	0.45 (0.27‐0.75)	.002	0.48 (0.28‐0.82)	.007
Smoke (yes vs no)	1.39 (0.43‐4.54)	.581	1.34 (0.39‐4.55)	.641
ER (+ vs −)	1.15 (0.66‐2.00)	.620	0.73 (0.26‐2.04)	.549
PR (+ vs −)	1.27 (0.76‐2.13)	.363	1.32 (0.60‐2.94)	.492
Her‐2 (+ vs −)	0.73 (0.44‐1.23)	.238	0.82 (0.47‐1.44)	.489
Surgery				
Biopsy only	1.00 (ref)		1.00 (ref)	
BCT	2.00 (0.44‐8.98)	.368	3.32 (0.31‐35.38)	.321
MRM	2.31 (0.49‐10.78)	.288	3.78 (0.21‐68.90)	.369
AJCC stage				
0‐1	1.00 (ref)		1.00 (ref)	
2	0.98 (0.59‐1.61)	.926	0.87 (0.47‐1.62)	.655
3‐4	0.91 (0.51‐1.62)	.746	0.88 (0.36‐2.17)	.785
Irradiated target side				
Right	1.00 (ref)		1.00 (ref)	
Left	0.77 (0.49‐1.21)	.256	0.75 (0.47‐1.21)	.241
Bilateral	0.76 (0.21‐2.77)	.671	0.62 (0.13‐2.97)	.551
CTV				
Breast only	1.00 (ref)		1.00 (ref)	
Chest wall only	2.12 (0.67‐6.72)	.201	2.27 (0.35‐14.82)	.394
Breast + SCF	0.74 (0.39‐1.42)	.365	0.89 (0.40‐1.98)	.768
Chest wall + SCF	1.03 (0.59‐1.77)	.924	0.92 (0.15‐5.73)	.931

BCS, breast conserving surgery; CI, confidence interval; CTV, clinical targeted volume; ER, estrogen receptors; HER‐2, human epidermal growth factor receptor‐2; MRM, modified radical mastectomy; N/A, not applicable; OR, odds ratio; PR, progesterone receptors; ref, reference group.

## 4 DISCUSSION

Previous research reported that providing additional RT boost to the resection cavity considerably improves local tumor control.^17^ Poor long‐term cosmetic outcomes and high complication rates were reported for highly inhomogeneous doses. In the case of daily doses of 2.5 Gy per fraction or doses above 50 Gy to the whole breast, some areas of the breast may receive inhomogeneous radiation doses.^18^ Numerous studies have been designed with the objective of reducing the adverse effects of acute radiation skin toxicity using hygiene regimes or creams; however, these studies have not presented an effective strategy for preventing skin reactions.^19^ To our knowledge, our study is the first retrospective randomized trial demonstrating that IGRT induces significantly lower acute radiation skin toxicity than IMRT does.

Vicini et al reported the first clinical use of breast IMRT and noted a low occurrence of acute adverse effects on the skin in 2002.^8^ Another study presented the results of a matched‐pair analysis of patients who had received either breast IMRT or standard RT.^9^ The study reported a significantly lower level of moist desquamation with IMRT than with standard RT using wedge compensation. In our study, comparing IGRT with IMRT revealed that patients subjected to IMRT exhibited considerably higher levels of moist desquamation than did those subjected to IGRT.

Previous studies have analyzed the setup error associated with an EPID or CBCT for breast cancer treatment. In one study,^20^ patients with breast cancer were treated to quantify the differences in setup errors associated with CBCT and an EPID. Another study^21^ showed that the ability to record patients’ patterns of breathing‐related movements during RT using pretreatment imaging techniques such as CBCT and the breath‐hold technique is another advantage of IGRT over IMRT. Thus, IGRT is a promising new RT technique that might considerably reduce the cardiac irradiation doses administered to patients with cancer of the left breast, thereby potentially reducing long‐term cardiac complications. An identical rationale was considered for radiation skin toxicity when the IGRT treatment modality was selected for the patients.

In our report, we demonstrate that the main objective of using IGRT with CBCT or MVCT with TOMO instead of IMRT in combination with an EPID for treating breast cancer was for reducing radiation‐induced skin toxicity. Our study revealed that IGRT with CBCT or TOMO induced relatively low radiation skin toxicity and focused on the severity of RD. The present study is the first report, globally, to suggest that IGRT causes considerably lower radiation skin toxicity than does IMRT.

In conclusion, IGRT including TOMO and IMRT combined with CBCT is a modality that can be used in different RT treatments. The results of this study demonstrate that IGRT induces considerably lower severity of acute RD than IMRT does when combined with an EPID. In the future, additional prospective studies should be conducted to verify and support this finding.

## CONFLICT OF INTEREST

The authors declare no conflict of interest.
